# Socioeconomic inequalities in teenage pregnancy in Nigeria: evidence from Demographic Health Survey

**DOI:** 10.1186/s12889-022-14146-0

**Published:** 2022-09-12

**Authors:** Chijioke Ifeanyi Okoli, Mohammad Hajizadeh, Mohammad Mafizur Rahman, Eswaran Velayutham, Rasheda Khanam

**Affiliations:** 1 School of Business, and Centre for Health Research, University of Southern Queensland, Toowoomba, QLD 4350 Australia; 2 Department of Health Administration and Management, Faculty of Health Sciences and Technology, College of Medicine, University of Nigeria, Enugu Campus, Enugu, Enugu State, Nigeria; 3 School of Health Administration, Dalhousie University, Halifax, Canada; 4 College for Indigenous Studies, Education and Research, University of Southern Queensland, Toowoomba, QLD 4350 Australia

**Keywords:** Teenage pregnancy, Socioeconomic inequalities, Concentration curve, Concentration index, Decomposition analysis, Nigeria

## Abstract

**Background:**

Despite the high rate of teenage pregnancy in Nigeria and host of negative medical, social and economic consequences that are associated with the problem, relatively few studies have examined socioeconomic inequality in teenage pregnancy. Understanding the key factors associated with socioeconomic inequality in teenage pregnancy is essential in designing effective policies for teenage pregnancy reduction. This study focuses on measuring inequality and identifying factors explaining socioeconomic inequality in teenage pregnancy in Nigeria.

**Methods:**

This is a cross sectional study using individual recode (data) file from the 2018 Nigeria Demographic Health Survey. The dataset comprises a representative sample of 8,423 women of reproductive age 15 – 19 years in Nigeria. The normalized Concentration index (C_n_) was used to determine the magnitude of inequalities in teenage pregnancy. The C_n_ was decomposed to determine the contribution of explanatory factors to socioeconomic inequalities in teenage pregnancy in Nigeria.

**Results:**

The negative value of the C_n_ (-0.354; 95% confidence interval [CI] = -0.400 to -0.308) suggests that pregnancy is more concentrated among the poor teenagers. The decomposition analysis identified marital status, wealth index of households, exposure to information and communication technology, and religion as the most important predictors contributing to observed concentration of teenage pregnancy in Nigeria.

**Conclusion:**

There is a need for targeted intervention to reduce teenage pregnancy among low socioeconomic status women in Nigeria. The intervention should break the intergenerational cycle of low socioeconomic status that make teenagers’ susceptible to unintended pregnancy. Economic empowerment is recommended, as empowered girls are better prepared to handle reproductive health issues. Moreover, religious bodies, parents and schools should provide counselling, and guidance that will promote positive reproductive and sexual health behaviours to teenagers.

## Plain language summary

Teenage pregnancy is a global public health concern. It is an undesirable occurrence and seems to be one of the social problems facing several countries, including Nigeria. Previous studies suggest socioeconomic differences in teenage pregnancy in Nigeria. However, relatively few studies have examined the socioeconomic inequality in teenage pregnancy in the country. This study focuses on measuring and explaining predictors of socioeconomic inequality in teenage pregnancy in Nigeria using the Nigeria Demographic Health Survey conducted in 2018. Findings suggest that teenage pregnancy is more concentrated among poor teenagers, while the most important factor contributing to the teenage pregnancy in Nigeria were marital status, wealth index of households, exposure to information and communication technology, and religion. These findings emphasise the need for targeted intervention to reduce teenage pregnancy among low socioeconomic status women in Nigeria to break the intergenerational cycle that expose teenagers to unwanted pregnancy. Since, empowered girls are better prepared to handle reproductive health issues. In addition, religious bodies, parents and schools should give teenagers counselling, and guidance that will promote positive reproductive and sexual health behaviours.

## Background

Approximately 21 million teenage girls aged 15–19 years become pregnant each year and the prevalence of teenage pregnancies is 95% higher in low- and middle-income countries (LMICs) compared with high-income countries [1]. Globally teenage pregnancy poses a profound public health concern [2–5]. For instance, pregnancy and childbirth complications are the major cause of death in teenage girls and 99% of all teenage maternal death occurs in LMICs [1, 6].

Teenage pregnancy constitutes a significant economic, health, and social cost to the mothers and newborn children, their families, and the wider society [4]. Specifically, early motherhood has far-reaching consequences including an increased risk of antenatal complications and mortality, failure to complete schooling, socioeconomic disadvantage, welfare dependence, marital difficulties, maternal depression and less competent parenting [7, 8]. Children born to teenage mothers have higher rates of health problems (preterm birth, low birth weight, intra-uterine growth retardation, neonatal death, etc.), physical injury, behavioural difficulties, cognitive problems, and educational underachievement compared to children born to the adult mothers [6, 8–10]. Indeed, teenage pregnancy is an undesirable phenomenon and seems to be one of the social problems facing several countries, including Nigeria [11].

In Nigeria, according to the National Population Commission, 23 percent of girls aged 15 to 19 years have started childbearing [12, 13]. About 400,000 unplanned births occur annually in Nigeria and half of these births are to teenage girls between the ages of 15 and 19 years [11]. Pregnant schoolchildren in Nigeria are often victims of ridicule in school, which forces them to drop out of school even before school authorities expel them for being pregnant [14].

The high rate of teenage pregnancy rate (106 adolescent births per 1000 population) is a major concern for the government and other stakeholders [2]. To reduce the unintended pregnancies among schoolchildren, a curriculum for sex education was introduced in Nigerian schools in 2002 [15]. However, the poor attitude of the teachers and inadequate support from parents and religious leaders has led to the failure to implement this curriculum [15]. Specifically, many policymakers, government officials, religious leaders and parents fear that talking about sex with young people will only encourage promiscuous behaviour [16]. In fact, none of the sex education mandates had made any significant contribution to the decline of teenage pregnancy [17, 18].

Studies suggest that there are geographical differences in teenage pregnancy in Nigeria [2, 12]. While every three adolescent/teenage girls in Northern Nigeria get pregnant, the corresponding figure is one out of ten girls in the South [12]. Also, teenage girls with lower levels of education, lower-income households and living in rural areas are more likely to experience adolescent pregnancy compared with those from high socioeconomic status (SES) backgrounds [3, 5, 12, 19].

Despite the high rate of teenage pregnancy and host of negative medical and socioeconomic consequences that are associated with the problem in Nigeria and sub-Saharan Africa in general, relatively few studies have examined the socioeconomic inequality in teenage pregnancy in the region [2–5, 19]. This study focuses on measuring and explaining predictors of socioeconomic inequality in teenage pregnancy in Nigeria. Understanding the key factors associated with socioeconomic inequality in teenage pregnancy is essential in designing effective policies in reducing teenage pregnancy [3]. This is particularly crucial given that the high teenage pregnancy rate in Nigeria and other African countries portends danger to the actualization of the Sustainable Development Goal 5 (i.e., achieve gender equality and empower all women and girls) by 2030.

## Methods

### Study area

The study area is Nigeria, with an estimated population of 198 million in 2018 [20]. About 70 percent of the population resides in rural areas while only about 30 percent lives in urban areas [21]. With 32.4 percent of the population below the age of 18 years and over 23% adolescents/teenagers [22, 23], Nigeria has a large youth population. Administratively, the country is divided into six geopolitical zones viz., North-Central, North-East, North-West, South-East, South-West, and South-South. Of the six geopolitical zones in Nigeria, southern states had the highest youth literacy rate while northern states had the least youth literacy rate [24]. Approximately 21.3 percent of youths, aged 15–19 had never been to school [24].

### Data source

The dataset for the analysis comprises women of reproductive age of 15–19 years in the six geopolitical zones of Nigeria. Data were obtained from the latest Nigeria Demographic Health Survey (NDHS), conducted between August 14, 2018 and December 29, 2018. DHS is conducted every five years with common questionnaires and/or variables that are generalizable to over 90 low- and middle-income countries [13]. The NDHS data is a representative of Nigerian population with a response rate of 99%. The study used Individual (women’s) Recode data file that collected information on women’s background characteristics, reproductive history, household asset ownership, etc. The NDHS uses a multistage sampling procedure, standardized tools and well-trained interviewers to collect reliable data on maternal and child health. The details of the survey are explained elsewhere [13].

### Sample

The sample size for the study was limited to 8,423 women (currently or ever pregnant) of reproductive age 15–19 years in Nigeria. As per DHS recommendation, sample weight was applied to get the representative sample size. The sample focused on the variable ‘currently or ever pregnant’ and “teenage current age” rather than “teenage age at first birth”.

### Variables

#### Outcome variable

The outcome variable in the study is teenage pregnancy. The variable is a dummy variable coded 1 if a teenager (aged 15–19 years) currently or ever pregnant, 0 otherwise.

#### Socioeconomic status

The socioeconomic status of a teenager was measured using wealth index as an indicator of socioeconomic status. Since information on individuals’ expenditure or income are often difficult to collect [25–27], the NDHS constucts a wealth index, as a measure of SES, using easy-to-collect data on a household ownership of selected assets (e.g., car, televisions and bicycles), materials used in housing construction, type of water access, and sanitation facilities [26]. A principal component analysis (PCA) technique was used to construct households’ wealth index scores based on the aforementioned information collected in the survey [13]. The first principal component of a set of variables captures the largest amount of information that is common to all the variables [25–27]. Households’ wealth index scores were used to categorise individuals into five SES quintile, starting with the poorest to the richest.

#### Independent variables

In line with previous literature [2, 3, 6, 12], the following variables were used as predictors of teenage pregnancy:, teenage education level, marital status, religion, occupation, place of residence, geopolitical zone, wealth index quintiles, and exposure to information and communication technology (ICT) (frequency of watching television and use of internet). Table 1 presents description of variables used in the study.

**Table 1:** Description of variables used in the study

Variable	Variable description
Currently or ever pregnant	
No	1 = if a teenager is not currently or have not been pregnant, 0 otherwise
Yes	1 = if a teenager is currently or have been pregnant, 0 otherwise
**Sociodemographic variables**	
* Teenage current age*	
Age 15	1 = if a teenager is 15 years old, 0 otherwise
Age 16	1 = if a teenager is 16 years old, 0 otherwise
Age 17	1 = if a teenager is 17 years old, 0 otherwise
Age 18	1 = if a teenager is 18 years old, 0 otherwise
Age 19	1 = if a teenager is 19 years old, 0 otherwise
* Marital status*	
Never married	1 = if a teenager is never married, 0 otherwise
Married	1 = if a teenager is married, 0 otherwise
* Ethnic origin*	
Hausa/Fulani/Kanuri	1 = if a teenager ethnic origin is Hausa/Fulani/Kanuri, 0 otherwise
Igbo	1 = if a teenager ethnic origin is Igbo, 0 otherwise
Yoruba	1 = if a teenager ethnic origin is Yoruba, 0 otherwise
Others	1 = if a teenager ethnic origin is not Hausa, Igbo or Yoruba, 0 otherwise
**Socioeconomic variables**	
* Teenage highest education level*	
No formal education	1 = if a teenager has no formal education, 0 otherwise
Primary education	1 = if a teenager has a primary education, 0 otherwise
Secondary education	1 = if a teenager has a secondary education, 0 otherwise
Higher	1 = if a teenager has a higher education, 0 otherwise
* Wealth index*	
Poorest	1 = if a teenager is in the poorest quintile, 0 otherwise
Poorer	1 = if a teenager is in poorer quintile, 0 otherwise
Middle	1 = if a teenager is in the middle quintile, 0 otherwise
Richer	1 = if a teenager is in richer quintile, 0 otherwise
Richest	1 = if a teenager is in the richest quintile, 0 otherwise
* Employment status*	
Unemployed	1 = if a teenager is not working, 0 otherwise
Employed	1 = if a teenager is working, 0 otherwise
* Religion*	
Christian	1 = if a teenager is a Christian, 0 otherwise
Muslim	1 = if a teenager is a Muslim, 0 otherwise
Others	1 = if a teenager is neither Christian nor Muslim, 0 otherwise
Geographic and geopolitical variables	
* Place of residence*	
Urban	1 = if a teenager lives in an urban area, 0 otherwise
Rural	1 = if a teenager lives in a rural area, 0 otherwise
* Geopolitical zone*	
North-Central	1 = if a teenager is from North-Central, 0 otherwise
North-East	1 = if a teenager is from North-East, 0 otherwise
North-West	1 = if a teenager is from North-East, 0 otherwise
South-East	1 = if a teenager is from South-East, 0 otherwise
South-South	1 = if a teenager is from South-South, 0 otherwise
South-West	1 = if a teenager is from South-West, 0 otherwise
**Exposure to information and communication technology (ICT)**	
* Frequency of watching television*	
Not at all	1 = if a teenager does not watch TV, 0 otherwise
Less than once a week	1 = if a teenager watches TV less than once a week, 0 otherwise
At least once a week	1 = if a teenager watches TV at least once a week, 0 otherwise
* Use of internet*	
No	1 = if a teenager does not use internet, 0 otherwise
Yes	1 = if a teenager uses internet, 0 otherwise

### Statistical analysis

#### Measuring socioeconomic inequalities in the teenage pregnancy

We used the concentration index (C) to measure socioeconomic inequality in teenage pregnancy. The C is measured based on the Concentration curve, which plots the cumulative share of health variables in horizontal axis against the cumulative share of population in ascending order of SES in the vertical axis. Twice the area between the Concentration curve and line of perfect equality (i.e., 45-degree line) indicate the magnitude of the C. If the Concentration curve lies above (or below) the line of perfect equality, it suggests that health outcome is concentrated among the poor (or rich).

The C was calculated using a convenient regression method as follows [28, 29]:

1 $$2{\sigma }_{r}^{2}\left(\frac{{h}_{i}}{\mu }\right)=\alpha +\beta {r}_{i}+{\varepsilon }_{i},$$

where ${\sigma }_{r}^{2}$ is the variance of the fractional rank, $h$ is the healthcare variable of interest (i.e., teenage pregnancy) of $i$ th teenage girl, $\mu$ is the mean of the health variable of interest, $h$, for the whole population, and ${r}_{i}=\frac{1}{N}$ is the fractional rank of the $i$ th teenage girl in the distribution of socioeconomic position, with $i=1$ for the poorest and $i=N$ for the richest teenager. The C is calculated as the ordinary least squares (OLS) estimate of $\beta$ [29, 30].

The C ranges from -1 to + 1, for continuous health outcomes. Since our health outcome variable of interest is binary, the minimum and maximum of the C are not between -1 and + 1 and depend on $\mu$ [31]. The C can be normalized by multiplying the estimated C by $\frac{1}{1-\mu }$ to overcome this issue. We used the normalized Concentration index (${C}_{n}$) to quantify socioeconomic inequalities in teenage pregnancy. If the value of the ${C}_{n}$ is zero, it suggests that there is no socioeconomic inequality in health outcomes. A negative (or positive) value of the ${C}_{n}$ indicates a higher concentration of the health variable among the poor (or rich) [28]. A higher value of the ${C}_{n}$ corresponds to higher socioeconomic inequality in health.

#### Decomposition analysis

In order to identify the contribution of each explanatory variable to socioeconomic inequality in teenage pregnancy, we decomposed the C_n_ using the Wagstaff, et al. approach [29]. Assume that we have a linear regression model to link our outcome variable (i.e., teenage pregnancy) $h$, to a set of $k$ explanatory factors, ${x}_{\kappa }$ such as:

2 $$h=\alpha +{\sum }_{\kappa }{\beta }_{\kappa }{x}_{\kappa }+ \varepsilon$$

where $\alpha$ is the intercept and $\beta$ denotes parameter that measure the relationship between each explanatory factor $x$ and the teenage pregnancy, and $\varepsilon$ is error term. A Wagstaff, E Van Doorslaer and N Watanabe [29] showed that the C of $h$ can be decomposed into the contribution of determinants that explain the teenage pregnancy as follows:

3 $$C={\sum }_{k}(\frac{{\beta }_{k}\overline{{\chi }_{k}}}{\mu }){C}_{K}+ \frac{{GC}_{\varepsilon }}{\mu },$$

where, $\overline{x }$_k_ is the mean of ${x}_{k}$, and ${C}_{k}$ denotes the C for${x}_{k}$, a contributing factor. The ${GC}_{\varepsilon }$ denotes the generalized C of the error term,${\varepsilon }_{i}$.

Equation 3 shows that the overall inequality in the teenage pregnancy has two components. The first term $(\frac{{\beta }_{k}\overline{{x }_{k}}}{\mu }){C}_{K}$ denotes the contribution of factor $k$ to socioeconomic inequality in the teenage pregnancy. It constitutes the deterministic or explained component of the teenage pregnancy of the C. The second term $\frac{{GC}_{\varepsilon }}{\mu }$ represents the unexplained component [28]. Based on Eq. 3, the product of the elasticity of each factor and its ${C}_{k}$ gives the contribution of that factor to the inequality. The negative (or positive) contribution of a predictor to the ${C}_{n}$ suggests that the socioeconomic distribution of the predictor and the association between the predictor and the teenage pregnancy leads to an increase in the concentration of teenage pregnancy among the poor (or rich). A zero value of either elasticity or the ${C}_{k}$ leads to the zero contribution of the factor to $C$ [28].

Applying the A Wagstaff [31] normalization approach to the decomposition of the $C$ can yield:

4 $${C}_{n}=\frac{C}{1-\mu }=\frac{{\sum }_{k}(\frac{{\beta }_{k}\overline{{x }_{k}}}{\mu }){C}_{K}}{1-\mu }+\frac{\frac{{GC}_{\varepsilon }}{\mu }}{1-\mu }$$

The dataset was weighted using the sampling weight provided in the NDHS to obtain estimates that are representative of all teenagers in Nigeria. Logit model estimation and marginal effects were conducted before the decomposition analysis. Chi-square was used to test associations between explanatory factors and teenage pregnancy. The predictors of teenage pregnancy were considered statistically significant at p < 0.05. All data analyses were conducted using Stata/SE-13 software [32].

## Results

### Descriptive statistics

Table 2 reports descriptive statistics of variables used in the study. About 6.3% of the teenagers are currently or ever pregnant and majority of them were never married (75.2%). The married teenagers (23.4%) were mainly from the Hausa/Fulani/Kanuri (43.0%) ethnic origin. In addition, 25.8% of the teenagers had no formal education, while 61.1% had secondary education. Over half of teenagers were Muslims (57.8%) and reside in rural areas (54.9%), in North-West (32.4%), North-East (17.7%) and North-Central (14.2%) geopolitical zones. On exposure to ICT, most teenagers did not use internet (84.4%), nor watch television at all (48.1%).

**Table 2:** Descriptive statistics of variables used in the study

Variable	Mean/percentage
Currently or ever pregnant (15-19yrs)	
No	93.7
Yes	6.3
**Sociodemographic variables**	
Teenage current age	
Age 15	24.6
Age 16	18.8
Age 17	18.7
Age 18	22.7
Age 19	15.2
* Marital status*	
Never married	75.2
Married	23.4
Others	1.4
* Ethnic origin*	
Hausa/Fulani/Kanuri	43.0
Igbo	13.9
Yoruba	13.2
Others	29.9
**Socioeconomic variables**	
* Teenage highest education level*	
No formal education	25.8
Primary education	10.4
Secondary education	61.1
Higher	2.7
* Wealth index*	
Poorest	16.9
Poorer	20.6
Middle	20.8
Richer	21.4
Richest	20.3
* Employment status*	
Unemployed	64.4
Employed	35.6
* Religion*	
Christian	41.7
Muslim	57.8
Others	0.5
**Geographic and geopolitical variables**	
* Place of residence*	
Urban	45.1
Rural	54.9
* Geopolitical zone*	
North-Central	14.2
North-East	17.7
North-West	32.4
South-East	10.9
South-South	10.5
South-West	14.3
**Exposure to information and communication technology (ICT)**	
* Frequency of watching television*	
Not at all	48.1
Less than once a week	19.4
At least once a week	32.5
* Use of internet*	
No	84.4
Yes	15.6

### Socioeconomic inequality in teenage pregnancy in Nigeria

**Fig. 1:**
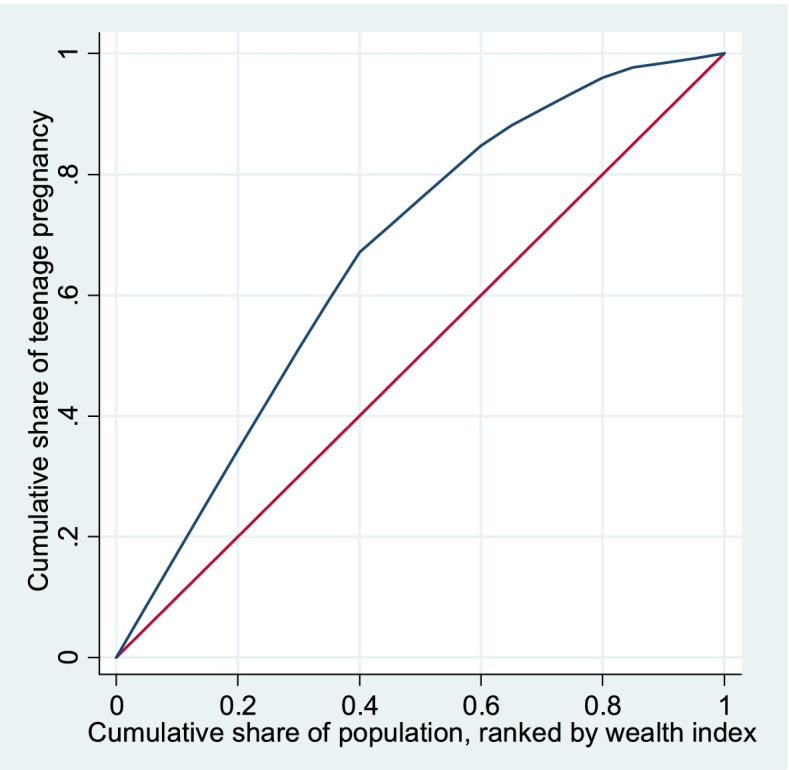
The Concentration curve of teenage pregnancy in Nigeria

Figure 1 presents the concentration curve of teenage pregnancy in Nigeria. The curve lies above the 45-degree diagonal line suggesting that teenage pregnancy in Nigeria is more concentrated among poor teenagers (C_n_ = -0.354; 95% CI = -0.400 to -0.308).

### Decomposition of the socioeconomic inequality in teenage pregnancy in Nigeria

Table 3 presents the decomposition of the socioeconomic inequality in teenage pregnancy in Nigeria. The table contains the estimated marginal effects of the predictor variables derived from the logit model, the elasticities, the C of the predictor variables (C_k_) and the contribution of each predictor variable to the C_n_. The elasticity column shows the change in the outcome variable (i.e., teenage pregnancy) associated with a one-unit change in the independent variables. It indicates the responsiveness of the health outcome (teenage pregnancy) to a change in the predictor variables. A negative (or positive) sign in elasticity shows a decreasing (or increasing) change of teenage pregnancy in association with a change in the predictor.

**Table 3:** Decomposition of the socioeconomic inequality in teenage pregnancy in Nigeria, 2018

**Variables**	**Marginal effect**	$\overline{{\varvec{X}} }$	**Elasticities**	**C**_**k**_	**Contribution to C**_**n**_		**Percentage contribution (%)**
					**Absolute**	**Summed**	
**Teenage current age**							
Age 15	-0.007	0.246	-0.027	-0.019	0.000		
Age 16	-0.002	0.188	-0.006	0.025	0.000		
Age 17	-0.006	0.187	-0.018	-0.007	0.000		
Age 18	0.007	0.227	0.025	-0.046	-0.001	-0.001	0.18
Age 19 (ref)							
**Marital status**							
Married	0.248*	0.234	0.921	-0.374	-0.323	-0.323	91.12
Never married (ref)							
**Ethnicity**							
Hausa/Fulani/Kanuri (ref)							
Igbo	0.068**	0.139	0.150	0.288	0.040		
Yoruba	0.012	0.132	0.025	0.428	0.010		
Others	0.007	0.299	0.033	0.008	0.000	0.051	-14.35
**Teenage highest education level**							
No formal education (ref)							
Primary	0.010	0.104	0.016	-0.258	-0.004		
Secondary	0.016*	0.611	0.155	0.227	0.033		
Tertiary	-0.012	0.027	-0.005	0.599	-0.003	0.026	-7.38
**Wealth index of households**							
Poorest (ref)							
Poorer	0.006	0.206	0.020	-0.456	-0.008		
Middle	0.004	0.208	0.013	-0.042	-0.001		
Richer	-0.004	0.214	-0.014	0.38	-0.005		
Richest	-0.023*	0.203	-0.074	0.797	-0.055	-0.069	19.50
**Employment status**							
Unemployed (ref)							
Employed	-0.015*	0.356	-0.085	-0.088	0.007	0.007	
**Religion**							
Christian (ref)							
Muslim	0.012	0.578	0.110	-0.138	-0.014		
Others	0.041	0.005	0.003	-0.204	-0.001	-0.015	4.19
**Place of residence**							
Urban	0.005	0.451	0.036	0.345	0.012	0.012	-3.27
Rural (ref)							
**Geopolitical zone**							
North-Central (ref)							
North-East	-0.005	0.177	-0.014	-0.292	0.004		
North-West	0.001	0.324	0.005	-0.206	-0.001		
South-East	-0.027	0.110	-0.047	0.225	-0.010		
South-South	0.002	0.105	0.003	0.319	0.001		
South-West	0.027	0.144	0.062	0.447	0.026	0.020	-5.58
**Frequency of watching television**							
Not at all (ref)							
Less than once a week	-0.019*	0.194	-0.058	0.144	-0.008		
At least once a week	-0.009	0.325	-0.046	0.416	-0.018	-0.026	7.34
**Use of internet**							
No (ref)							
Yes	-0.012	0.156	-0.030	0.564	-0.016	-0.016	4.43
**Sum**						-0.334	94.23
**Residual**						-0.020	5.77
**Total C**_**n**_						-0.354	100.00

Marginal effects were calculated at the means of the predictor. The percentage of contributions was calculated by dividing the specific “summed” contribution by the absolute values of C_n_ and multiplying by 100. The sum of all the percentage contributions should add up to 100 percent. The value 0.00 is not zero, but due to rounding

^*^p < 0.005, **p < 0.1

The negative (or positive) sign of the C_k_ for a certain variable indicates that the predictor concentrated among the poor (or rich) teenagers. For instance, in Table 3, being married, primary education, employed, North-East and North-West geopolitical zones were concentrated among the poor, whereas, the teenage secondary and tertiary highest education levels, urban residence, southern geopolitical zones, exposure to ICT (frequency of watching TV, and use of internet) were more concentrated among the rich.

The estimated contribution of predictors to the C_n_ suggested that marital status, primary and tertiary education, wealth index of households, religion, geopolitical zones (North-East and South-East) and frequency of watching TV and use of internet contributed negatively to socioeconomic inequality in teenage pregnancy in 2018 in Nigeria. On the other hand, ethnicity, secondary education level, place of residence, and southern geopolitical zones positively contributed to the socioeconomic inequality of teenage pregnancy in the country.

**Fig. 2:**
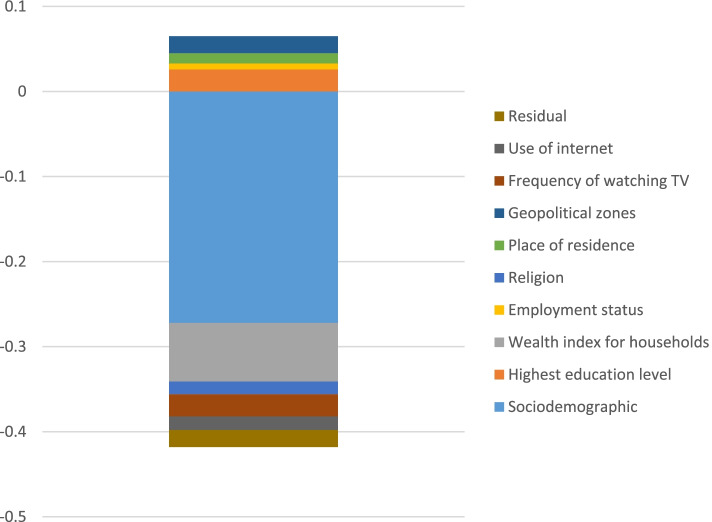
Absolute contribution of each factor to socioeconomic inequality in teenage pregnancy in Nigeria, 2018. The y-axis shows the absolute negative or positive contribution of each predictor to the C_n_

Figure 2 illustrates the absolute contribution of a predictor to the socioeconomic inequality of teenage pregnancy in 2018 in Nigeria. As reported in Table 3 and illustrated in Fig. 2, marital status (91.1%), wealth index of household (19.5%), frequency of watching TV (7.3%), use of internet (4.4%), and religion (4.2%) were the most important predictors contributing to or explained the observed socioeconomic inequality in teenage pregnancy in Nigeria. In contrast, ethnicity (-14.4%), teenage education level (-7.4%), geopolitical zones (-5.6%) and place of residence (-3.3%) contributed negatively to socioeconomic inequality in teenage pregnancy.

The results show that the independent variables included in the model explained a sum of 94.2% of the observed socioeconomic inequality in teenage pregnancy in Nigeria. A 5.8% lower contribution of residual component suggests that there are less significant predictors other than the variables in the model that affect teenage pregnancy in Nigeria, which could not be identified by this study.

## Discussion

Understanding the predictors of the observed socioeconomic inequality in teenage pregnancy is vital in designing effective policies [3]. As the high teenage pregnancy rate in Nigeria portends danger to the actualization of SDG 5.3 by 2030, this study aimed at measuring and identifying factors associated with socioeconomic inequality in teenage pregnancy in Nigeria using the Concentration index approach.

The key findings show teenage pregnancy in Nigeria is more concentrated among the poor teenagers. The finding is in tandem with studies in Malawi [3] and Tanzania [5] that teenage pregnancy and childbearing rate was higher among teenagers from poorer household than those from richer households. It also aligns with the finding, which states that teenagers from low SES background are twice more likely to get pregnant as a teenager when compared with those from high SES background [19].

Findings further indicate that the most important predictors contributing to observed concentration of teenage pregnancy among the low SES in Nigeria were marital status, wealth index of households, frequency of watching TV, use of internet, and religion. This finding suggests that low SES (high level of poverty) makes teenagers susceptible to early pregnancy [2]. It is also traceable to the prevailing cultural norms and religious practices that prohibit teenagers from accessing family planning services and having premarital sex [2]. More so, access to reproductive health products is constrained by social stigma [33], thereby leading to unintended pregnancies. Hence, interventions sensitive to religious beliefs and cultural peculiarities should be designed to tackle the challenges of teenage pregnancies among the poor [19]. Religious leaders may set the pace through moral instructions in churches and mosques [11], coupled with counselling and guidance on risk associated with teenage pregnancy.

Our findings show over half of the teenagers are Muslims residing in rural areas in the northern geopolitical zones of the country. Study indicates that girls in the poorest wealth quintile are 2.5 times more likely to get married in childhood than those living in the richest quintile [34]. This is more prevalent among girls who live in rural areas than their urban counterparts due to economic, social, cultural, and religious factors [35]. Often marriage attracts a dowry for the bride’s family and in Nigeria, there is a real economic incentive for early marriage owing to dismal economic circumstances and strong cultural traditions in the region [35]. Even, young girls residing in rural areas are forcefully married because parents believe that it would save their daughters from sexual abuse [6]. Early marriage is one of the main contributors to teenage pregnancy among the low SES. Measures to delay age at marriage can help reduce early pregnancies. Therefore, eliminating child marriage needs to be part of family planning campaigns to facilitate the attainment of SDG 5.3 by 2030 [35]. This is important because teenagers who are at the risk of becoming pregnant is the relevant population from a policy viewpoint [36].

There is a need for formal education empowerment to address the high prevalence of teenage pregnancy among low SES girls in Nigeria. Empowered girls or women are more likely to delay marriage, and plan their pregnancies [34, 37]. Study shows that teenagers with higher education were 94% less likely to experience teenage pregnancy compared to those without education [12]. Also, it is a fact that teenagers who have higher education levels are protected from unwanted pregnancies due to the empowerment that accompany higher education [2]. Indeed, education plays a vital role given that those with low or no education tend to fall victim of early pregnancy compared to those who acquired higher education [12]. Given the high proportion of teenage pregnancy in the northern part of the country, due to low-level of education and high level of poverty, a targeted formal education intervention is warranted. It would help break the intergenerational cycle of poverty [37] and reduce the risk of early marriage, and teenage pregnancy [35].

The main strength of this paper is the use of the concentration index to identify key predictors of the observed socioeconomic inequality in teenage pregnancy in Nigeria. In addition, the paper used the latest NDHS 2018 dataset, which is nationally representative and generalizable to Nigeria as a whole. A major limitation of this paper is that, we cannot establish temporality between explanatory factors and socioeconomic inequality in teenage pregnancy given the cross-sectional design of the study. Thus, it precludes establishing causal inference. Further, since the survey data was self-reported, the issue of recall bias and social desirability may occur.

## Conclusion

There is a need for targeted intervention (i.e. increased girls’ enrolment and completion of high education), especially in the northern geopolitical zones of Nigeria. This may help break the intergenerational cycle of poverty that make teenagers’ susceptible to unintended pregnancy and parents forcing the girl-child into early marriage. Economic and education empowerment is recommended, as empowered girls/women are better prepared to handle reproductive health issues. Moreover, religious bodies, parents and schools should provide counselling, and guidance that will promote positive reproductive and sexual health behaviours to teenagers.

## Data Availability

Data for this research is publicly accessible from the DHS program archive after due permission and can be download from https:// www.dhsprogram.com/data/available-datasets.cfm.

## Abbreviations

C: Concentration index
FMoH: Federal ministry of health
ICT: Information and communication technology
LMICs: Low- and middle-income countries
NBS: National bureau of statistics
NDHS: Nigeria demographic health survey
NPC: National population commission
OLS: Ordinary least square
PCA: Principal component analysis
SDG: Sustainable development goal
SES: Socioeconomic status
WHO: World health organization
